# Brain and Hepatic *Mt* mRNA Is Reduced in Response to Mild Energy Restriction and *n*-3 Polyunsaturated Fatty Acid Deficiency in Juvenile Rats

**DOI:** 10.3390/nu9101145

**Published:** 2017-10-19

**Authors:** Aaron A. Mehus, Matthew J. Picklo, Sr

**Affiliations:** USDA Agricultural Research Service Grand Forks Human Nutrition Research Center, Grand Forks, ND 58203, USA; matthew.picklo@ars.usda.gov

**Keywords:** metallothionein, cerebellum, energy restriction, mRNA, juvenile, brain development, *n*-3 PUFA

## Abstract

Metallothioneins (MTs) perform important regulatory and cytoprotective functions in tissues including the brain. While it is known that energy restriction (ER) and dietary *n*-3 polyunsaturated fatty acid (PUFA) deficiency impact postnatal brain growth and development, little data exist regarding the impact of undernutrition upon MT expression in growing animals. We tested the hypothesis that ER with and without dietary *n*-3 PUFA deficiency reduces MT expression in juvenile rats. ER rats were individually pair-fed at 75% of the ad libitum (AL) intake of control rats provided diets consisting of either soybean oil (SO) that is α-linolenic acid (ALA; 18:3*n*-3) sufficient or corn oil (CO; ALA-deficient). Fatty acids (FA) and metal concentrations of liver and brain regions were analyzed. Tissue expression of MTs (*Mt1-3*) and modulators of MT expression including glucocorticoid receptors (*Nr3c1* and *Nr3c2*) and several mediators of thyroid hormone regulation (*Dio1-3*, *Mct8*, *Oatp1c1*, *Thra*, and *Thrb*) were measured. Plasma corticosterone and triiodothyronine levels were also evaluated. ER, but not metal deficiency, reduced *Mt2* expression in the cerebellum (50%) and cerebral cortex (23%). In liver, a reduction in dietary *n*-3 PUFA reduced *Mt1*, *Mt2*, *Nr3c1*, *Mct8*, and *Thrb*. ER elevated *Nr3c1*, *Dio1*, and *Thrb* and reduced *Thra* in the liver. Given MT’s role in cellular protection, further studies are needed to evaluate whether ER or *n*-3 PUFA deficiency may leave the juvenile brain and/or liver more susceptible to endogenous or environmental stressors.

## 1. Introduction

Undernutrition is a global problem that impedes brain and body development during childhood. Infants and children are particularly susceptible to undernutrition because of the high energy demands required for normal growth [1]. Childhood stunting affects approximately 162 million children <5 years of age worldwide [2]. Stunting has several lasting effects on children, including reduced physical stature, declined learning ability, psychological deficits, and elevated infection rates [2]. Similarly, energy restriction (ER) in juvenile animal models modifies neuronal growth, disrupts energy balance, induces behavioral changes, and decreases the immune response [3,4,5,6]. 

Dietary fat intake is important to infants and children during development. It is well-established that the long chain *n*-3 polyunsaturated fatty acid (*n*-3 LCPUFA) docosahexaenoic acid (DHA, 22:6*n*-3) preferentially accumulates in the brain. Within the brain, DHA is incorporated into the cell membranes where it promotes brain development and manifests influential effects on learning, memory, and behavior [7]. DHA needs to be consumed directly or is produced indirectly through its precursor α-linolenic acid (ALA; 18:3*n*-3). *n*-3 PUFA deficiency may have several deleterious effects within the brain including altering immune response, increasing susceptibility to oxidative damage, modifying genes involved in neuronal growth, and disturbing energy regulation [7,8,9,10].

In the brain, metallothionein isoforms (MTs) provide important roles in cytoprotection and the regulation of cellular metal ion concentration. The downregulation of brain MTs increases the likelihood of cellular oxidative stress and damage associated with heavy metal exposure [11]. Moreover, MT-1/2-deficient mice are more susceptible to neuronal damage associated with kainic acid (KA)-induced seizures [12]. In mammals, MT-1 and MT-2 (MT-1/2) expression is ubiquitous, whereas MT-3 is mainly localized to the brain but is expressed to a lesser degree in other organs [13,14,15]. MT-4 expression is largely confined to stratified epithelial cells associated with skin, esophagus, and the stomach [16]. Brain MT-1/2 expression is highly enriched in astrocytes, while MT-3 is observed in both astrocytes and neurons. MTs have not been reported to be expressed in oligodendrocytes or microglia [17]. 

MTs are highly conserved, low-molecular-weight, cysteine-rich proteins. The high cysteine content of MTs enables them to regulate metal ion homeostasis, particularly zinc and copper. Through this mechanism, MTs regulate metal-dependent cellular processes such as gene transcription, translation, and enzyme activity [11]. MT expression is regulated by metals, oxidative stress, glucocorticoids, inflammatory mediators, and thyroid hormone (TH) [17,18].

Little information is available in regard to the nutritional regulation of MTs. In older animals, several studies have reported an increased expression of *Mt1* and *Mt2* in response to ER in various tissues including those of the cerebellum, cortex, hippocampus, striatum, cochlea, spinal cord, heart, lung, skeletal muscle, and colon [19,20,21]. Moreover, *Mt3* along with several other antioxidant genes are upregulated in mammary glands of rats fed a low-fat (16% fat energy) *n*-3 PUFA-enriched diet compared to a high-fat (39% fat energy) *n*-3 PUFA-enriched diet [22]. In mice, MTs protect against development of obesity, insulin resistance, and diet-induced oxidative stress damage [23,24,25,26].

Given the neurotoxicological importance of MT expression in the postnatal brain, we explored the degree to which ER and dietary *n*-3 PUFA deficiency modifies MT expression. We tested the hypothesis that ER and dietary *n*-3 PUFA deficiency reduce MT expression in juvenile rats. Tissue concentrations of metals and the expression of glucocorticoid receptors and mediators of TH regulation along with plasma corticosterone (CORT) and triiodothyronine (free T3 or fT3) were studied as secondary endpoints given their ability to regulate MT levels and responsiveness to ER [18,27,28,29]. Fatty acid (FA) analysis was performed to confirm tissue *n*-3 PUFA depletion. Our data demonstrate that expression of *Mt2* decreased 50% in an energy-dependent manner within the cerebellum, while *Mt1*, *Mt2*, *Nr3c1*, *Mct8,* and *Thrb* decreased in an *n*-3 FA-dependent fashion in the liver. ER increased *Nr3c1*, *Dio1*, and *Thrb* and decreased *Thra* in the liver. Plasma CORT was elevated in the SO-ER diet compared to ad libitum levels, while fT3 was unchanged.

## 2. Materials and Methods 

### 2.1. Animals

All experiments were performed in accordance with the NIH guidelines for the use of live animals and were approved by the Institutional Animal Care and Use Committee of the USDA Agricultural Research Service, Grand Forks Human Nutrition Research Center. The animal experimentation and diets have been previously described [10]. Male Sprague–Dawley rats (21 days old) were purchased from Harlan Laboratories. Two days after arrival, rats were equally distributed, based on body mass, into four diet groups (*n* = 8/group): diets containing soybean oil (SO) with replete α-linolenic acid (ALA; 18:3*n*-3; 1.1% energy) with ad libitum (AL) intake (SO-AL), SO with ER (SO-ER), corn oil (CO; ALA-deficient; 0.13% energy) with AL intake (CO-AL), and CO with ER feeding (CO-ER). The SO and CO diets had linoleic acid (LA; 18:2*n*-6) at 8.6% energy and 8.8% energy, respectively. Oils constituted 16% fat energy. SO-ER and CO-ER rats were individually fed 75% of the diet consumed against a corresponding rat in the AL group receiving their respective dietary oil. All diets were based on the AIN93G formula [30]. SO-ER and CO-ER diets contained 25% more mineral and vitamin mix than the respective SO-AL and CO-AL diets to limit confounding factors resulting from micro-nutrient deficiency. 

Vitamin-free casein (Harlan/Teklad) was used as a protein source. CO and SO were purchased from Dyets Inc. All diets contained AIN93 mineral mix (Dyets Inc., Bethlehem, PA, USA) and AIN93G vitamin mix (Harlan/Teklad). Mineral analysis was also performed on the diets. Caloric content of the diets was determined by bomb calorimetry as described previously [31]. Food consumption was measured daily, and fresh food was provided daily. Body mass was measured weekly. Body composition was determined by whole-body MRI (EchoMRI-700; Echo Medical Systems LLC) at the beginning and end of the study. 

Rats remained in their dietary groups for 4 weeks. At the end of the experiment, rats were fasted overnight. Rats were killed by an overdose of anesthetic (xylazine 13 mg/kg and ketamine 100 mg/kg) followed by exsanguination. Blood was collected with EDTA for subsequent isolation of plasma. Brains were dissected into the cerebral cortex, hippocampus, and cerebellum. Brain regions, liver, and plasma were snap-frozen in liquid nitrogen and stored at −80 °C. Tissues were pulverized using a BioPulverizer (Biospec Products, Inc., Bartlesville, OK, USA) at liquid nitrogen temperature. Pulverized samples were stored at −80 °C until further use.

### 2.2. FA Analyses

A powdered sample of tissue (∼50 mg) was extracted twice into 3.6 mL of hexane/isopropanol (3:2 *v*/*v*) with BHT (50 μM; Sigma-Aldrich, St. Louis, MO, USA) added to limit lipid peroxidation. Samples were homogenized using a PRO200 Bio-Gen Series homogenizer (PRO Scientific Inc., Oxford, CT, USA) and then centrifuged at 2000× *g* for 10 min. The organic phase was removed, dried under nitrogen, and dissolved in 1 mL of hexane/2-propanol (3:2 *v*/*v*) containing 5% water and 50 μM BHT and stored at −80 °C under nitrogen. The FA content of the organic extract was determined by fatty acid methyl ester (FAME) analysis [32].

### 2.3. Gene Expression Analysis

RNA was isolated from pulverized tissue, ~20 mg, using a QIAcube system (Qiagen) as described previously [31,32]. qPCR was performed as described previously [32]. Primer pairs for gene expression are presented in Appendix A, Table A1.

### 2.4. Mineral Analysis 

Pulverized tissues were lyophilized, weighed, and digested with an acid reflux until solubilized and then analyzed by inductively coupled argon plasma emission spectroscopy (ICP) or inductively coupled argon plasma mass spectrometry (ICPMS). We included standard reference materials (SRMs) during all steps of our analysis, attempting to match the sample’s matrix when possible. The SRM used for tissue (cerebellum, cerebral cortex, and liver) was bovine Liver-SRM 1577b (NIST, Gaithersburg, MD, USA). Blanks were run periodically to assure there was no cross-contamination from one sample to another. Liver mineral analysis was performed via ICP using a Thermo Fisher Scientific ICAP 6500 Duo (Thermo Fisher Scientific, Waltham, MA, USA) as previously described [33]. The wavelengths used to analyze the multiple elements using ICP are reported in Appendix A. Analysis of cerebellum and cerebral cortex was performed by ICPMS using a Nexion 350 D (PerkinElmer Inc., Waltham, MA, USA). Analysis for the following elements was measured at the following assigned mass:
ElementMinimum Detection Limit^63^Cu0.63 ng/mL^55^Mn0.33 ng/mL^66^Zn2.61 ng/mL

### 2.5. Plasma CORT and fT3

CORT (Enzo Life Sciences, Farmingdale, NY, USA) and free triiodothyronine (fT3) (Genway Biotech, San Diego, CA, USA) concentrations in EDTA plasma were determined using commercially available enzyme immunoassay kits according to the manufacturer’s instructions. The inter-assay coefficient of variation (CV) for CORT and fT3 was <10% and <5%, respectively. 

### 2.6. Statistical Analyses

Two-way ANOVA was performed on all data sets using GraphPad Prism version 7.00 for Windows, GraphPad Software, La Jolla, CA, USA, www.graphpad.com. Tukey’s multiple comparison tests were performed when a significant interaction was identified (energy X oil). Significance was taken as *p* ≤ 0.05. 

## 3. Results

### 3.1. Energy Intake and Body Composition 

**Figure 1:**
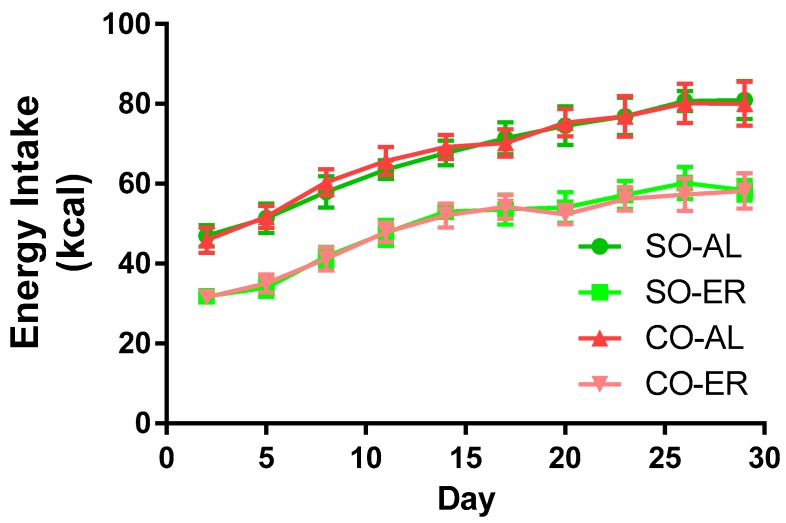
Energy intake of the experimental groups. Growing male rats at 3 weeks of age were fed AIN93G-based diets formulated to provide 16% of energy as fat derived from SO or CO. SO-ER and CO-ER rats were individually fed 75% of the diet amount of the SO-AL and CO-AL rats, respectively. For clarity of the graph, data are presented for every 3rd day on diet. Comparisons of food consumption by two-way ANOVA demonstrated no effect of oil type. Data are reported as mean ± SD; *n* = 8. AL: ad libitum; CO: corn oil; ER: energy restriction; SO: soybean oil.

Energy intake data are provided in Figure 1. Oil type did not influence energy intake in the AL or in the ER groups. Energy intake was 25% lower in the ER animals compared to the AL animals. The body composition data are presented in Table 1. By the end of the 4-week study, rats in the ER groups had body masses that were ~24% lower than those in the AL groups. There was no influence of oil type on body mass. Liver mass was reduced by ~15% and ~17% in ER animals fed the SO and CO diets, respectively. Total brain mass was not altered from *n*-3 PUFA depletion, but the CO-ER animals had slightly reduced brain mass compared with the CO-AL animals (~6%). ER animals had an elevated brain/body mass ratio regardless of oil type.

### 3.2. ER and n-3 PUFA-Deficient Diet Effects on Mt1-3 mRNA Expression in the Cerebellum, Cerebral Cortex, Hippocampus, and Liver 

The extent to which ER and reduced dietary *n*-3 PUFA modify *Mt1-3* gene expression in multiple brain regions and the liver was examined (Table 2). In the cerebellum, both ER groups had reduced *Mt1* (≥20%) and *Mt2* (50%) expression compared to the ad libitum groups, while expression of *Mt3* was unchanged. ER also reduced *Mt2* expression in the cerebral cortex (20%) and hippocampus (15%), but had no effect on *Mt1* and *Mt3* expression. *Mt2* expression was elevated (28%) in the cerebellum of CO-ER compared to SO-ER animals. *Mt1* gene expression was reduced (~15%) in the cerebral cortex of animals receiving the CO diet. 

*n*-3 PUFA depletion decreased *Mt1-2* expression in the liver >50%; however, ER had no effect on *Mt1-2* mRNA content in the liver. *Mt3* mRNA in the liver was not considered expressed since the ΔCt values were much higher compared with *Mt1* and *Mt2* (Appendix A, Table A2). This finding coincides with several previous reports of *Mt3* gene and protein expression being absent in mouse and rat liver [14,15,34,35]. The qPCR Ct values for β-*actin* that were used for target gene normalization are reported in Appendix A, Table A5.

### 3.3. Impact of the Low-n-3 FA Diet and ER on Liver PUFA Content

Because oil-induced changes in hepatic Mt gene expression were observed, we measured hepatic PUFA content. Consumption of the CO diet versus the SO diet reduced the hepatic content of ALA (92%), docosapentaenoic acid *n*-3 (DPA*n*-3; 22:5*n*-3) (49%), and DHA (51%) (Table 3). Hepatic content of eicosapentaenoic acid (EPA; 20:5*n*-3) was below the limit of detection in animals consuming the CO diet. ER reduced the hepatic concentration of ALA (51%) but not that of other n-3 PUFAs as previously reported [10]. 

Hepatic *n*-6 PUFA concentrations were modified by both oil types and ER. ALA restriction reduced hepatic LA content (14%). Intake of the low *n*-3 PUFA CO diet increased levels of docosatetraenoic acid (DTA; 22:4*n*-6) (149%). Hepatic content of docosapentaenoic acid *n*-6 (DPA*n*-6; 22:5*n*-6) was elevated greater than 20-fold by intake of the CO diet. Intake of the CO diet decreased dihomo-gamma-linolenic acid (DGLA; 20:3*n*-6) by 23%, while ER increased DGLA by 103%.

### 3.4. Impact of Low-n-3 FA Diet and ER on Cerebellar PUFA

Table 4 displays the cerebellar PUFA concentrations. Intake of the low-ALA CO diet elevated the concentration of arachidonic acid (ARA; 20:4*n*-6) (10%), DTA*n*-6 (16%), and DPA*n*-6 (520%), but reduced the amount of DGLA (14%). DPA*n*-6 was elevated by ER (30%). There were reductions in both DHA (10%) and DPA*n*-3 (~50%) in the CO groups.

### 3.5. Impact of Low-n-3 FA Diet and ER on Cerebral Cortex PUFA

The extent to which ER and a low *n*-3 PUFA diet affected PUFA levels in the cerebral cortex is reported in Table 5. ER elevated the concentration of ARA (7%), DPA*n*-6 (15%), and DHA (12%). Intake of the low *n*-3 PUFA CO diet increased the levels of DTA (25%) and DPA*n*-6 (230%), but reduced the level of DPA*n*-3 (30%). There were reductions in both LA (15%) and DGLA (12%) in the CO groups. This latter finding is likely the result of the elevated formation of the *n*-6 LCPUFA DTA and DPA*n*-6. 

### 3.6. Copper, Manganese, and Zinc Levels in the Cerebellum, Cerebral Cortex, and Liver

MT tissue expression can be induced by exposures to essential metals, primarily zinc, copper, and manganese [36]. In order to determine whether ER or oil-dependent reductions in the tissue concentrations of these metals occurred, these metals were analyzed in the cerebellum, cerebral cortex, and liver by either ICP or ICP-MS. In the cerebellum and liver, the levels of all 3 metals were unchanged from ER (Table 6). Copper content was unchanged in all tissues. Hepatic manganese content was slightly increased (8%) from intake of the CO diet. Interestingly, the level of zinc in the cerebral cortex of the SO-AL group was lower (~36%) than the other three groups. Mineral analysis was not performed on the hippocampus due to the low sample amount. 

### 3.7. Plasma CORT and fT3 Levels 

Because MTs are regulated by both glucocorticoids and THs [18,29], we determined plasma concentrations of CORT and fT3. Plasma CORT concentration was elevated 140% in the SO-ER compared to SO-AL group and 47% by the CO-ER diet compared to the CO-AL diet. fT3 concentrations were not changed between the dietary groups (Table 7).

### 3.8. ER and n-3 PUFA-Deficient Diet Effects on Glucocorticoid and Mineralocorticoid mRNA Expression in the Cerebellum, Cerebral Cortex, Hippocampus, and Liver 

MT mRNA and protein levels are induced by glucocorticoids and ER increases glucocorticoid receptor (GR) protein in mice. We analyzed the mRNA levels of the glucocorticoid and mineralocorticoid receptors (*Nr3c1* and *Nr3c2*, respectively). There were no ER or *n*-3 PUFA effects on the mRNA levels of either gene in any of the brain regions analyzed (Table 8). However, in the liver, SO-ER and CO-ER diets increased *Nr3c1* expression (78% and 62%, respectively) from the ad libitum groups. Hepatic *Nr3c1* gene expression was reduced 18% by dietary *n*-3 PUFA deficiency. Appendix A, Table A3, shows the ΔCt values for *Nr3c1* and *Nr3c2*.

### 3.9. ER and n-3 PUFA-Deficient Diet Effects on mRNA Expression of TH Regulators in the Cerebellum, Cerebral Cortex, Hippocampus, and Liver

We analyzed mRNA expression for several regulators of TH signaling including: TH activation/inactivation by type I, II, and III iodothyronine deiodinases (*Dio1-3*), TH transport by monocarboxylate transporter 8 (*Mct8*) and solute carrier organic anion transporter family member 1C1 (*Oatp1c1*), and thyroid hormone receptors alpha (*Thra*) and beta (*Thrb*). Appendix A, Table A4, displays the ΔCt values for *Dio1-3*, *Mct8*, *Oatp1c1*, *Thra*, and *Thrb* throughout the tissues analyzed in this study.

There was no change of *Dio1* expression in the three brain regions among the dietary treatments (Table 9). The expression of *Dio2* and *Dio3* was increased 13% and 32%, respectively, in the cerebellum from ER. *Oatp1c1* was decreased (23%) in the CO-ER compared to CO-AL groups in the cortex. ER elevated *Thra* (9%) and *Thrb* (19%) mRNA concentrations in the cerebral cortex. Similarly, cerebellar *Thra* expression was elevated (12%) from ER.

Expression of *Dio2* was elevated (38%) in the cerebral cortex of the low *n*-3 PUFA CO-AL diet animals compared to SO-AL animals. *Mct8* was decreased from *n*-3 PUFA deficiency in the cerebellum (60%) *Oatp1c1* displayed an oil-dependent reduction in the cerebellum (28%) and cortex (21%). Cerebellar *Thra* and *Thrb* were decreased (10% and 14%, respectively) from dietary *n*-3 PUFA depletion, whereas increases in *Thra* and *Thrb* expression from *n*-3 PUFA depletion (~20%) occurred in the cerebral cortex.

Hepatic *Dio1* expression for the SO-ER and CO-ER groups was elevated (67% and 87%, respectively) from the ad libitum groups. In the liver, *Thra* was decreased in response to SO-ER and CO-ER diets (43% and 39%, respectively) compared to the ad libitum groups. *Thrb* was increased in the liver by SO-ER and CO-ER diets (62% and 66%, respectively) compared to the ad libitum groups. TH transporter expression in the liver was not impacted by ER.

Dietary *n*-3 PUFA effects on liver TH regulators were minor. *Mct8* expression was decreased by *n*-3 PUFA deficiency in the liver (26%). Hepatic *Thrb* was reduced (19%) in the *n*-3 PUFA-deficient group (CO-AL) compared to SO-AL. 

## 4. Discussion

Undernutrition in children is a persistent global issue. Specifically, the developing cerebellum is particularly sensitive to undernutrition and is susceptible to environmental insults [37,38]. The cerebellum is best known for regulating motor coordination but plays critical roles in higher cognitive functions [39,40]. ER in juvenile animals, not unlike undernutrition in children, can disrupt brain growth, function, and behavior [3,4,5,6]. 

Reducing dietary ALA is used in animal models to deplete brain DHA [41,42]. In the current study, there was no additive effect to hepatic or cerebellar DHA loss when ER is combined with *n*-3 PUFA depletion. These findings correlate with our previous results using this juvenile undernutrition model [10].

The most prominent discoveries reported here were the >50% reduction of *Mt2* in the cerebellum from ER and >50% reduction of *Mt1-2* in the liver from *n*-3 PUFA depletion. The ER-dependent alterations of *Mt-2* expression we report oppose the response, which has been described in aging animals. In older animals, microarray experiments have shown elevated *Mt1* and *Mt2* expression in response to ER in several rodent tissues [19,20,21]. The elevation of MTs by ER in older animals is not entirely clear, but may be a response in which MTs are regulating free radical activity, oxidative stress, apoptotic activity, and metal homeostasis. This notion is supported by the fact that overexpression of MTs increases the mouse lifespan [43]. 

In certain areas of the rat brain, *Mt1* and *Mt3* mRNA decrease in response to a zinc-deficient diet [44]. The downregulation of cerebellar *Mt2* mRNA in the ER groups of our study does not appear to be a metal-dependent response given that there was no decrease in zinc, copper, or manganese concentrations in the analyzed tissues. This observation was not unexpected since we increased the vitamin and mineral content of the diets fed to the rats undergoing ER to compensate for the reduced energy intake. This finding is evidence that a metal-independent mechanism regulates *Mt2* expression within the cerebellum of juvenile rats undergoing ER. 

Several studies demonstrate that MTs are induced by glucocorticoids through a GR-mediated mechanism [13,45,46,47,48]. The secretion of glucocorticoids is a well-known endocrine response to stress, and glucocorticoids bind to either the GR (*Nr3c1*) or mineralocorticoid receptor (MR, or *Nr3c2*), both of which are ubiquitously expressed. Our results agree with those published demonstrating that ER in juvenile and adult rats increases plasma CORT concentrations, the main rodent glucocorticoid [4,28,49]. Thus, the ER-dependent decreases in *Mt1-2* expression are not the result of decreased plasma CORT concentrations. We did not observe a clear transcriptional relationship of *Mt2* levels in the cerebellum compared with *Nr3c1* or *Nr3c2* levels. While we did detect an ER-dependent increase and an *n*-3 PUFA-dependent reduction in hepatic *Nr3c1,* the impact of these changes is equivocal. 

While THs (thyroxine, T4, and/or triiodothyronine, T3) are best known for their functions in regulating energy metabolism and immune responses, they play important roles in brain and cerebellar development [50,51]. Previous reports demonstrate that *Mt3* mRNA is reduced by thyroxine in the developing rat brain [18]. Moreover, MT mRNA and protein levels are inducible by thyroid-stimulating hormone (TSH), and their induction is considered a thyroid stress marker of autoimmune diseases [52,53]. Studies in mammals report that ER decreases TH (T3 and/or T4) in serum or plasma [54,55]. It has also been demonstrated that type 1 iodothyronine deiodinase (D1, *Dio1* gene) mRNA and enzyme activity is decreased in rodent livers from ER [55,56]. The most prominent ER-dependent modifications we observed were increases in *Dio1* and its mediator *Thrb* mRNA concentrations within the livers of the ER groups and may be part of a compensatory mechanism to regulate TH levels under ER in our model. Further studies are necessary to determine if *Dio1-3*, *Thra*, *Thrb*, *Oatp1c1*, or *Mct8* play a role in regulating *Mt1-2* expression in the cerebellum or liver during ER and/or *n*-3 PUFA deficiency.

The decrease in cerebellar *Mt1-2* and increase in liver *Dio1* as well as the unchanged plasma concentrations of fT3 in response to ER are inconsistent with the previous studies described above [21,54,56]. However, there are important factors to take into consideration that may help explain these discrepancies. The previous studies were initiated in adult or ageing animals, whereas our study was initiated in juvenile animals (weanling rats). The other important factors are the durations and extent of ER. We employed a mild (75% of ad libitum) 4-week dietary ER supplemented with vitamins to compensate for any effects that micro/macro-nutrients might have had. On the other hand, several ER studies utilize anywhere between 35 and 50% ER without additional vitamins and the duration of ER can vary immensely. In general, the onset of ER (juveniles vs. adults), the extent of ER (25–50%), and the duration of ER (acute vs. chronic) are influencing factors on the physiological outcomes of ER in mammals [57].

A limitation of this study is the lack of protein levels for the MT isoforms. Their small size, high cysteine level, high sequence similarity, and large number of isoforms limits the effectiveness of traditional antibody-based methods to accurately quantify each MT isoform at the protein level. However, a previous study reported that *Mt1* mRNA in the cerebellum and other brain areas correlate to MT-1/2 protein by radioimmunoassay methods in rats in response to glucocorticoids [13].

The hepatic *n*-3 PUFA-dependent reduction in *MT1-2* may be explained in part through a mechanism that involves the generation of oxidized lipids such as cyclopentenones and multiple alkenals like 4-hydroxy-2-hexenal that are formed from the oxidation of DHA and EPA [58]. Cyclopentenones and 4-hydroxyalkenals are bioactive molecules capable of activating the antioxidant response element (ARE) pathway through nuclear factor erythroid 2-related factor 2 (Nrf2) and potentially Nrf1 [58,59]. *Mt1-2* contain ARE binding sites within their promoter region, and data suggest that *Mt1-2* expression is elevated in an ARE/Nrf1-dependent manner [60,61]. This decrease in hepatic levels of *MT1-2* from *n*-3 PUFA depletion in our juvenile undernutrition rat model may indicate an increased vulnerability to the hepatoxic effects associated with infections, drugs, or heavy metals against which MTs provide protection [62,63,64,65].

Subsequent studies that include multiple time points are needed to fully characterize the effects of ER on cerebellar *Mt2* expression since the exact regulatory mechanism is still unclear. MTs have clear neurotoxicological and neuropathological importance. Within the brain, MTs provide neuroprotection, and several studies have demonstrated their importance in the brain injury repair process. Brain MTs are elevated in response to ischemia, bacterial lipopolysaccharides (LPS), traumatic brain injury, seizures, and psychological stress [17]. This response of brain MTs is thought to be driven by oxidative stress and neuroinflammation. Accordingly, MTs are frequently elevated in several neuropathological conditions associated with inflammation such as Alzheimer’s disease, amyotrophic lateral sclerosis (ALS), multiple sclerosis (MS), and lysosomal storage disorders (LSD) [17,66]. If MT levels in the cerebellum are permanently modified from the ER in juveniles, this may increase their susceptibility to infection, heavy metal exposures, or other stressors that could potentially impede normal brain development leading to lifelong adverse health problems.

## 5. Conclusions

This study enhances the limited knowledge base pertaining to the biological effects of undernutrition in juvenile animals. More specifically, we analyzed *Mt* expression alterations in a postnatal developmental rat model undergoing ER and/or *n*-3 PUFA depletion. Further studies are needed to assess the possible health and toxicological outcomes associated with the cerebellar and hepatic downregulation of a gene (*Mt*) that serves several important functions within tissues. 

## Figures and Tables

**Table 1:** Body composition in growing male rats.

					*p*		
Endpoint	SO-AL	SO-ER	CO-AL	CO-ER	Energy	Oil	Energy X Oil
**Body Mass (g)**							
Begin	54.6 ± 1.7	54.7 ± 1.6	54.5 ± 1.7	54.3 ± 2.4	0.92	0.72	0.83
End	258.4 ± 9.4	197.5 ± 7.5	251.0 ± 10.8	192.3 ± 10.0	**<0.01**	0.07	0.75
**Liver Mass (g)**	7.6 ± 0.4	6.5 ± 0.7	7.2 ± 0.8	6.0 ± 0.7	**<0.01**	0.07	0.71
**Brain Mass (g)**	1.6 ± 0.1	1.6 ± 0.1	1.7 ± 0.1	1.6 ± 0.1	**0.01**	0.82	0.38
**Brain/Body (%)**	0.63 ± 0.03	0.80 ± 0.04	0.66 ± 0.02	0.81 ± 0.06	**<0.01**	0.25	0.65

Data are reported as mean ± SD, *n* = 8. Two-way ANOVA was performed for each parameter. Bold indicates significance, *p* < 0.05. AL: ad libitum; CO: corn oil; ER: energy restriction; SO: soybean oil.

**Table 2:** Tissue expression of metallothionein mRNA.

	mRNA Fold-Change Compared with SO-AL				*p*		
Tissue/Gene	SO-AL	SO-ER	CO-AL	CO-ER	Energy	Oil	Energy X Oil
**Cerebellum**							
*Mt1*	1.02 ± 0.18	0.82 ± 0.19	0.99 ± 0.26	0.75 ± 0.12	**<0.01**	0.44	0.79
*Mt2*	1.11 ± 0.61	0.50 ± 0.12	1.34 ± 0.45	0.64 ± 0.11	**<0.01**	**0.04**	0.97
*Mt3*	1.03 ± 0.29	0.92 ± 0.12	1.02 ± 0.22	0.84 ± 0.14	0.06	0.48	0.51
**Cerebral Cortex**							
*Mt1*	1.00 ± 0.13	0.87 ± 0.21	0.83 ± 0.21	0.76 ± 0.14	0.10	**0.03**	0.57
*Mt2*	1.02 ± 0.27	0.77 ± 0.17	0.88 ± 0.32	0.70 ± 0.12	**0.02**	0.15	0.62
*Mt3*	1.00 ± 0.09	0.96 ± 0.09	0.94 ± 0.11	0.91 ± 0.09	0.23	0.10	0.86
**Hippocampus**							
*Mt1*	1.01 ± 0.11	0.96 ± 0.15	1.04 ± 0.16	1.06 ± 0.12	0.72	0.18	0.42
*Mt2*	1.01 ± 0.12	0.84 ± 0.16	1.03 ± 0.22	0.86 ± 0.10	**<0.01**	0.69	0.75
*Mt3*	1.00 ± 0.10	1.04 ± 0.11	0.94 ± 0.09	0.99 ± 0.06	0.21	0.08	0.81
**Liver**							
*Mt1*	1.08 ± 0.40 ^A^	1.01 ± 0.44 ^A^	0.47 ± 0.24 ^B^	0.73 ± 0.18 ^A,B^	0.16	**<0.01**	**0.05**
*Mt2*	1.09 ± 0.48	1.05 ± 0.58	0.44 ± 0.27	0.70 ± 0.22	0.21	**<0.01**	0.07
*Mt3* *†*	-	-	-	-	-	-	-

Data are reported as mean ± SD, *n* = 8. Two-way ANOVA was performed on the initial ΔCt values for each gene. Labeled means in a row without a common superscript letter differ, *p* ≤ 0.05. Bold indicates significance. AL: ad libitum; CO: corn oil; ER: energy restriction; *Mt1-3*: metallothionein 1-3; SO: soybean oil; ΔCt: change in cycle threshold normalized to *β-actin*; † signifies that the gene is not considered expressed in this tissue (ΔCt values reported in Appendix A, Table A1).

**Table 3:** Polyunsaturated fatty acid (PUFA) concentration in the liver of juvenile male rats.

	Tissue Concentration, µmol/g				*p*		
FAs	SO-AL	SO-ER	CO-AL	CO-ER	Energy	Oil	Energy X Oil
***n*-6 PUFAs**							
18:2	21.33 ± 4.26	18.88 ± 4.14	18.29 ± 4.57	15.46 ± 3.29	0.08	**0.03**	0.90
18:3	0.30 ± 0.10	0.30 ± 0.07	0.26 ± 0.06	0.25 ± 0.12	0.88	0.18	0.87
20:2	0.34 ± 0.06	0.38 ± 0.08	0.34 ± 0.08	0.30 ± 0.14	0.95	0.26	0.22
20:3	0.31 ± 0.05 ^A^	0.63 ± 0.18 ^B^	0.24 ± 0.06 ^A^	0.37 ± 0.18 ^A^	**<0.01**	**<0.01**	**0.04**
20:4	21.98 ± 3.32	23.11 ± 8.00	25.47 ± 5.94	23.66 ± 4.41	0.87	0.32	0.47
22:4	0.39 ± 0.08	0.39 ± 0.13	0.97 ± 0.19	0.98 ± 0.19	0.92	**<0.01**	0.92
22:5	0.10 ± 0.11	0.19 ± 0.09	2.23 ± 0.62	2.46 ± 0.57	0.29	**<0.01**	0.67
*n*-6 LCPUFAs ^1^	23.10 ± 3.53	24.70 ± 8.41	29.26 ± 6.48	27.77 ± 5.23	0.98	**0.04**	0.49
***n*-3 PUFAs**							
18:3	0.87 ± 0.37 ^A^	0.43 ± 0.15 ^B^	0.07 ± 0.05 ^C^	0.02 ± 0.03 ^C^	**<0.01**	**<0.01**	**0.01**
20:5	0.26 ± 0.18	0.30 ± 0.06	BDL	BDL	0.54	**<0.01**	0.49
22:5	1.06 ± 0.19 ^A^	1.42 ± 0.55 ^A^	0.54 ± 0.13 ^B^	0.39 ± 0.20 ^B^	0.34	**<0.01**	**0.03**
22:6	6.71 ± 1.04	7.11 ± 2.65	3.26 ± 0.88	2.91 ± 0.46	0.97	**<0.01**	0.49
*n*-3 LCPUFAs ^2^	8.03 ± 1.23	8.83 ± 3.20	3.80 ± 1.00	3.30 ± 0.65	0.82	**<0.01**	0.32

Data are reported as mean ± SD, *n* = 8. Two-way ANOVA was performed for each parameter. Labeled means in a row without a common superscript letter differ, *p* ≤ 0.05. Bold indicates significance. ^1^ *n*-6 LCPUFAs sums all *n*-6 PUFAs except 18:2*n*-6 and 18:3*n*-6. ^2^ *n*-3 LCPUFAs sums all *n*-3 PUFAs except 18:3*n*-3. AL: ad libitum; BDL: below detection level; CO: corn oil; ER: energy restriction; FA: fatty acid; LCPUFA: long-chain polyunsaturated fatty acid; SO: soybean oil.

**Table 4:** PUFA concentration in the cerebellum of juvenile male rats.

	Tissue Concentration, µmol/g				*p*		
FAs	SO-AL	SO-ER	CO-AL	CO-ER	Energy	Oil	Energy X Oil
***n*-6 PUFAs**							
18:2	1.90 ± 0.14	1.81 ± 0.20	1.65 ± 0.10	2.08 ± 1.49	0.53	0.97	0.34
20:2	0.55 ± 0.07	0.53 ± 0.10	0.52 ± 0.06	0.51 ± 0.05	0.63	0.30	0.89
20:3	0.68 ± 0.05	0.65 ± 0.10	0.61 ± 0.07	0.56 ± 0.03	0.08	**<0.01**	0.71
20:4	9.19 ± 0.58	9.06 ± 0.80	9.69 ± 0.52	9.93 ± 0.72	0.81	**<0.01**	0.44
22:4	2.92 ± 0.22	2.84 ± 0.35	3.38 ± 0.19	3.26 ± 0.18	0.27	**<0.01**	0.80
22:5	0.27 ± 0.11	0.35 ± 0.03	1.68 ± 0.24	1.93 ± 0.20	**<0.01**	**<0.01**	0.16
*n*-6 LCPUFAs ^1^	13.61 ± 0.78	13.43 ± 1.26	15.87 ± 0.94	16.18 ± 0.79	0.84	**<0.01**	0.48
***n*-3 PUFAs**							
22:5	0.27 ± 0.03	0.25 ± 0.02	0.14 ± 0.02	0.15 ± 0.04	0.25	**<0.01**	0.20
22:6	15.11 ± 1.16	15.13 ± 1.12	13.67 ± 0.70	14.07 ± 0.63	0.53	**<0.01**	0.57
*n*-3 LCPUFAs ^2^	15.38 ± 1.19	15.37 ± 1.11	13.82 ± 0.71	14.22 ± 0.61	0.56	**<0.01**	0.54

Data are reported as mean ± SD, *n* = 8. Two-way ANOVA was performed for each parameter with significance as *p* ≤ 0.05. Bold indicates significance. ^1^ *n*-6 LCPUFAs sums all *n*-6 PUFAs except 18:2*n*-6. ^2^ *n*-3 LCPUFAs sums all *n*-3 PUFAs. AL: ad libitum; CO: corn oil; ER: energy restriction; FA: fatty acid; LCPUFA: long-chain polyunsaturated fatty acid; SO: soybean oil.

**Table 5:** PUFA concentration in the cerebral cortex of juvenile male rats.

	Tissue Concentration, µmol/g				*p*		
FAs	SO-AL	SO-ER	CO-AL	CO-ER	Energy	Oil	Oil X Energy
***n*-6 PUFAs**							
18:2	1.23 ± 0.11	1.19 ± 0.09	1.06 ± 0.07	1.01 ± 0.08	0.21	**<0.01**	0.93
20:2	0.18 ± 0.02	0.18 ± 0.02	0.19 ± 0.02	0.19 ± 0.03	0.95	0.39	0.73
20:3	0.41 ± 0.04	0.42 ± 0.04	0.36 ± 0.03	0.38 ± 0.05	0.28	**<0.01**	0.93
20:4	12.08 ± 0.75	12.93 ± 0.59	12.77 ± 1.19	13.42 ± 0.72	**0.02**	0.06	0.74
22:4	3.20 ± 0.23	3.45 ± 0.09	4.01 ± 0.41	4.19 ± 0.43	0.06	**<0.01**	0.73
22:5	0.72 ± 0.04	0.83 ± 0.09	2.37 ± 0.37	2.66 ± 0.18	**0.01**	**<0.01**	0.23
*n*-6 LCPUFAs ^1^	16.59 ± 1.01	17.82 ± 0.63	19.69 ± 1.90	20.84 ± 1.23	**0.01**	**<0.01**	0.92
***n*-3 PUFAs**							
22:5	0.21 ± 0.03	0.23 ± 0.02	0.15 ± 0.02	0.15 ± 0.02	0.25	**<0.01**	0.40
22:6	14.32 ± 1.29	15.59 ± 0.92	14.53 ± 1.27	15.19 ± 1.50	**0.04**	0.84	0.50
*n*-3 LCPUFAs ^2^	14.53 ± 1.31	15.82 ± 0.93	14.67 ± 1.29	15.34 ± 1.50	**0.04**	0.71	0.49

Data are reported as mean ± SD, *n* = 8. Two-way ANOVA was performed for each parameter with significance as *p* ≤ 0.05. Bold indicates significance. ^1^ *n*-6 LCPUFAs sums all *n*-6 PUFAs except 18:2*n*-6. ^2^ *n*-3 LCPUFAs sums all *n*-3 PUFAs. AL: ad libitum; CO: corn oil; ER: energy restriction; FA: fatty acid; LCPUFA: long-chain polyunsaturated fatty acid; SO: soybean oil.

**Table 6:** Tissue mineral analysis.

					*p*		
Mineral (µg/g)	SO-AL	SO-ER	CO-AL	CO-ER	Energy	Oil	Energy X Oil
**Cerebellum**							
Copper	7.8 ± 3.6	9.9 ± 2.7	7.7 ± 1.2	8.8 ± 1.7	0.08	0.54	0.56
Manganese	2.6 ± 1.6	2.2 ± 0.5	2.2 ± 0.3	2.0 ± 0.3	0.27	0.28	0.79
Zinc	34.5 ± 10.7	51.9 ± 21.9	39.6 ± 6.0	39.1 ± 5.7	0.07	0.40	0.06
**Cerebral Cortex**							
Copper	10.3 ± 5.4	10.1 ± 1.5	11.6 ± 3.8	12.0 ± 3.9	0.97	0.25	0.85
Manganese	2.2 ± 0.5	1.8 ± 0.2	2.0 ± 0.4	1.9 ± 0.2	0.07	0.58	0.09
Zinc	48.7 ± 11.2 ^A^	77.7 ± 11.2 ^B^	77.2 ± 11.3 ^B^	73.8 ± 12.5 ^B^	**<0.01**	**<0.01**	**<0.01**
**Liver**							
Copper	15.5 ± 2.2	14.9 ± 3.9	15.2 ± 2.0	17.1 ± 2.0	0.50	0.31	0.19
Manganese	9.0 ± 0.8	8.9 ± 0.9	9.7 ± 0.6	9.7 ± 0.7	0.82	**<0.01**	0.86
Zinc	107.7 ± 10.1	107.8 ± 13.4	113.8 ± 7.3	112.2 ± 5.1	0.82	0.13	0.81

Data are reported as mean ± SD, *n* = 8. Two-way ANOVA was performed for each parameter. Labeled means in a row without a common superscript letter differ, *p* ≤ 0.05. Bold indicates significance. AL: ad libitum; CO: corn oil; ER: energy restriction; SO: soybean oil.

**Table 7:** Plasma concentrations of CORT and fT3.

					*p*		
Analyte	SO-AL	SO-ER	CO-AL	CO-ER	Energy	Oil	Energy X Oil
**CORT** (ng/mL)	51.6 ± 25.7	125.6 ± 42.7	89.9 ± 34.2	132.3 ± 79.6	**<0.01**	0.21	0.38
**fT3** (pg/mL)	2.5 ± 0.3	2.9 ± 0.4	2.8 ± 0.3	2.7 ± 0.3	0.16	0.51	0.06

Data are reported as mean ± SD, *n* = 8. Two-way ANOVA was performed for each parameter with significance as *p* ≤ 0.05. Bold indicates significance. AL: ad libitum; CO: corn oil; CORT: corticosterone; ER: energy restriction; SO: soybean oil; T3: triiodothyronine.

**Table 8:** Tissue expression of glucocorticoid and mineralocorticoid receptor mRNA.

	mRNA Fold-Change Compared with SO-AL				*p*		
Tissue/Gene	SO-AL	SO-ER	CO-AL	CO-ER	Energy	Oil	Energy X Oil
**Cerebellum**							
*Nr3c1*	1.05 ± 0.33	1.07 ± 0.34	0.78 ± 0.16	0.96 ± 0.21	0.29	0.10	0.33
*Nr3c2*	1.01 ± 0.17	1.10 ± 0.12	1.19 ± 0.18	1.15 ± 0.19	0.58	0.07	0.26
**Cerebral Cortex**							
*Nr3c1*	1.06 ± 0.41	1.07 ± 0.36	1.03 ± 0.21	1.02 ± 0.26	0.96	0.92	0.80
*Nr3c2*	1.03 ± 0.24	1.07 ± 0.25	1.13 ± 0.24	1.29 ± 0.23	0.22	0.06	0.57
**Hippocampus**							
*Nr3c1*	1.00 ± 0.08	1.02 ± 0.12	1.00 ± 0.14	0.94 ± 0.17	0.17	0.81	0.08
*Nr3c2*	1.30 ± 0.66	1.43 ± 0.34	1.75 ± 0.24	1.61 ± 0.17	0.49	0.06	0.27
**Liver**							
*Nr3c1*	1.02 ± 0.20	1.81 ± 0.65	0.84 ± 0.10	1.36 ± 0.30	**<0.01**	**0.01**	0.68
*Nr3c2*	1.25 ± 0.76	1.59 ± 0.54	1.28 ± 0.52	1.53 ± 0.50	0.17	0.67	0.76

Data are reported as mean ± SD, *n* = 8. Two-way ANOVA was performed on the initial ΔCt values for each gene with significance as *p* ≤ 0.05. Bold indicates significance. AL: ad libitum; CO: corn oil; ER: energy restriction; *Nr3c1*: nuclear receptor subfamily 3 group C member 1; *Nr3c2*: nuclear receptor subfamily 3 group C member 2; SO: soybean oil; ΔCt: change in cycle threshold normalized to *β-actin*.

**Table 9:** Tissue mRNA expression of thyroid hormone (TH) mediators.

	mRNA Fold-Change Compared with SO-AL				*p*		
Tissue/Gene	SO-AL	SO-ER	CO-AL	CO-ER	Energy	Oil	Energy X Oil
**Cerebellum**							
*Dio1*	1.03 ± 0.26	1.09 ± 0.30	0.97 ± 0.23	1.02 ± 0.27	0.70	0.54	0.91
*Dio2*	1.02 ± 0.20	1.15 ± 0.15	1.00 ± 0.18	1.07 ± 0.12	**0.05**	0.41	0.62
*Dio3*	1.08 ± 0.43	1.23 ± 0.23	0.85 ± 0.20	1.12 ± 0.32	**0.04**	0.18	0.68
*Mct8*	1.16 ± 0.76	1.31 ± 1.25	1.02 ± 0.60	0.52 ± 0.16	0.24	**0.05**	0.25
*Oatp1c1*	1.02 ± 0.18	0.98 ± 0.18	0.90 ± 0.20	0.71 ± 0.19	0.11	**<0.01**	0.25
*Thra*	1.00 ± 0.06	1.04 ± 0.09	0.90 ± 0.05	1.01 ± 0.11	**0.02**	**0.03**	0.23
*Thrb*	1.10 ± 0.64	0.94 ± 0.13	0.77 ± 0.10	0.81 ± 0.14	0.87	**0.02**	0.48
**Cerebral Cortex**							
*Dio1*	1.61 ± 1.45	1.85 ± 1.31	3.20 ± 3.70	1.39 ± 0.92	0.93	0.59	0.31
*Dio2*	1.01 ± 0.12^A^	1.21 ± 0.21^A,B^	1.39 ± 0.15^B^	1.30 ± 0.26^B^	0.39	**<0.01**	**0.03**
*Dio3*	1.07 ± 0.37	0.75 ± 0.50	1.33 ± 1.18	1.56 ± 0.92	0.58	0.12	0.19
*Mct8*	1.03 ± 0.30	1.01 ± 0.23	1.16 ± 0.38	1.25 ± 0.28	0.63	0.10	0.57
*Oatp1c1*	1.00 ± 0.09	0.96 ± 0.18	0.99 ± 0.19	0.76 ± 0.15	**0.01**	**0.05**	0.10
*Thra*	1.00 ± 0.07	1.09 ± 0.10	1.20 ± 0.13	1.28 ± 0.15	**0.05**	**<0.01**	0.77
*Thrb*	1.01 ± 0.14	1.20 ± 0.21	1.23 ± 0.15	1.32 ± 0.16	**0.02**	**<0.01**	0.33
**Hippocampus**							
*Dio1*	1.51 ± 1.83	1.96 ± 1.25	1.75 ± 1.51	1.55 ± 1.24	0.62	0.98	0.44
*Dio2*	1.01 ± 0.11	1.11 ± 0.13	1.03 ± 0.15	1.07 ± 0.19	0.21	0.84	0.53
*Dio3*	1.08 ± 0.42	1.41 ± 0.82	1.75 ± 1.42	1.49 ± 0.83	0.74	0.36	0.45
*Mct8*	1.05 ± 0.34	1.14 ± 0.48	1.15 ± 0.24	1.09 ± 0.33	0.99	0.65	0.50
*Oatp1c1*	1.03 ± 0.29	1.07 ± 0.22	1.04 ± 0.14	1.08 ± 0.15	0.58	0.68	0.97
*Thra*	1.01 ± 0.16	1.05 ± 0.15	1.12 ± 0.17	1.15 ± 0.15	0.47	0.08	0.94
*Thrb*	1.01 ± 0.12	1.18 ± 0.24	1.14 ± 0.32	1.29 ± 0.31	0.13	0.39	>0.99
**Liver**							
*Dio1*	1.03 ± 0.26	1.72 ± 0.41	0.82 ± 0.19	1.53 ± 0.37	**<0.01**	0.07	0.61
*Dio2* *†*	-	-	-	-	-	-	-
*Dio3*	1.19 ± 0.68	1.44 ± 2.41	0.59 ± 0.46	0.78 ± 0.69	0.85	0.12	0.32
*Mct8*	1.04 ± 0.30	1.06 ± 0.26	0.77 ± 0.22	0.79 ± 0.22	0.72	**<0.01**	0.96
*Oatp1c1* †	-	-	-	-	-	-	-
*Thra*	1.01 ± 0.12	0.58 ± 0.06	0.93 ± 0.12	0.57 ± 0.09	**<0.01**	0.31	0.50
*Thrb*	1.01 ± 0.13	1.64 ± 0.18	0.82 ± 0.08	1.36 ± 0.23	**<0.01**	**<0.01**	0.99

Data are reported as mean ± SD, *n* = 8. Two-way ANOVA was performed on the initial ΔCt values for each gene. Labeled means in a row without a common superscript letter differ, *p* ≤ 0.05. Bold indicates significance. AL: ad libitum; CO: corn oil; *Dio1-3*: deiodinase 1-3; ER: energy restriction; *Mct8*: monocarboxylate transporter 8; *Oatp1c1*: solute carrier organic anion transporter family member 1C1; SO: soybean oil; *Thra*: thyroid hormone receptor alpha; *Thrb*: thyroid hormone receptor beta; ΔCt: change in cycle threshold normalized to *β-actin*. † signifies that the gene is not considered expressed in this tissue.

## Appendix A

**ICP Wavelengths Used to Analyze Mineral Content of Diets and Liver Tissue:**

Element	Wavelength	Minimum Detection Limit
Ca	315.887 nm	101 ppb
Cu	324.752 nm	10.4 ppb
Fe	259.939 nm	11 ppb
Mg	279.077 nm	131 ppb
Mn	257.610 nm	11.3 ppb
P	214.914 nm	784 ppb
Zn	213.857 nm	16 ppb
Sc	361.383 nm	used as the internal standard
K	766.491 nm	3.328 ppm
Na	588.995 nm	2.037 ppm

**Table A1:** qPCR primer sequences.

Gene	Forward Primer	Reverse Primer
*β-actin*	GTG TGG ATT GGT GGC TCT ATC	CAG TCC GCC TAG AAG CAT TT
*Dio1*	TAC GGG CAA GGT GCT AAT G	GTC TGC TGC CTT GAA TGA AAT C
*Dio2*	GAA TGC CAC CTT CTT GAC TTT G	CTT GGT TCC GGT GCT TCT TA
*Dio3*	CGC GAC GTT GAC TTC CTT AT	ATA GGC ACC ATA TGC GGA AC
*Mct8*	CAG GAG GCA AAC CAG GAA TAT C	TGG CTG CAA ACA CCA CTA TC
*Mt1*	CCA GAT CTC GGA ATG GAC CCC AAC	GTG CAC TTG TCC GAG GCA CCT TTG
*Mt2*	GCG ATC TCT CGT TGA TCT CC	CAG GAG CAG GAT CCA TCT GT
*Mt3*	TGC CCC TGT CCT ACT GGT GGT TCC	CGC CTT TGC AAA CAC AGT CCT TGG
*Nr3c1*	GGG ACA CGA ATG AGG ATT GTA A	CAG AGG TAC TCA CAC CAT GAA C
*Nr3c2*	GAG GAC CAC TGA GAA CAA CTA C	GGA CCT GTG ACC ATT CTC TTT
*Oatp1c1*	GGG CAG GTG TCA GAA AGA TAA	TGA AAG CAT GAG AGT CGT ACA G
*Thra*	CGG TTA TCA CTA CCG CTG TAT C	CAC CCG CTT TGA ATC GTC TA
*Thrb*	GGC ATG TCT CTG TCG TCT TT	CGT CAC CTT CAT CAG GAG TTT

**Table A2:** ΔCt values for Mt1-3.

	ΔCT			
Tissue/Gene	SO-AL	SO-ER	CO-AL	CO-ER
**Cerebellum**				
*Mt1*	6.33 ± 0.27	6.65 ± 0.33	6.38 ± 0.37	6.76 ± 0.22
*Mt2*	4.87 ± 0.68	5.89 ± 0.36	4.51 ± 0.48	5.52 ± 0.28
*Mt3*	0.95 ± 0.37	1.08 ± 0.19	0.95 ± 0.30	1.21 ± 0.23
**Cerebral Cortex**				
*Mt1*	6.27 ± 0.18	6.51 ± 0.36	6.58 ± 0.34	6.70 ± 0.27
*Mt2*	5.52 ± 0.32	5.93 ± 0.31	5.78 ± 0.52	6.05 ± 0.27
*Mt3*	1.88 ± 0.13	1.95 ± 0.14	1.98 ± 0.17	2.03 ± 0.14
**Hippocampus**				
*Mt1*	6.54 ± 0.16	6.62 ± 0.22	6.50 ± 0.22	6.47 ± 0.18
*Mt2*	5.51 ± 0.16	5.79 ± 0.27	5.50 ± 0.31	5.73 ± 0.16
*Mt3*	2.08 ± 0.14	2.03 ± 0.15	2.17 ± 0.13	2.10 ± 0.08
**Liver**				
*Mt1*	−1.08 ± 0.62	−0.95 ± 0.73	0.19 ± 0.78	−0.60 ± 0.35
*Mt2*	0.18 ± 0.67	0.35 ± 0.91	1.64 ± 1.00	0.76 ± 0.46
*Mt3**†*	15.67 ± 0.59	15.45 ± 0.80	16.05 ± 0.63	14.69 ± 1.56

Data are reported as mean ± SD, *n* = 8. AL: ad libitum; CO: corn oil; ER: energy restriction; *Mt1-3*: metallothionein 1-3; SO: soybean oil; ΔCt: change in cycle threshold normalized to *β-actin*. † signifies that the gene is not considered expressed in this tissue.

**Table A3:** ΔCt values for glucocorticoid and mineralocorticoid receptors.

	ΔCt			
Tissue/Gene	SO-AL	SO-ER	CO-AL	CO-ER
**Cerebellum**				
*Nr3c1*	11.10 ± 0.46	11.09 ± 0.51	11.49 ± 0.33	11.19 ± 0.32
*Nr3c2*	6.81 ± 0.24	6.68 ± 0.16	6.57 ± 0.21	6.62 ± 0.24
**Cerebral Cortex**				
*Nr3c1*	9.32 ± 0.49	9.29 ± 0.46	9.30 ± 0.28	9.34 ± 0.38
*Nr3c2*	7.48 ± 0.36	7.40 ± 0.30	7.33 ± 0.30	7.13 ± 0.26
**Hippocampus**				
*Nr3c1*	6.31 ± 0.12	6.28 ± 0.17	6.21 ± 0.17	6.42 ± 0.26
*Nr3c2*	6.40 ± 1.40	5.92 ± 0.37	5.60 ± 0.23	5.71 ± 0.15
**Liver**				
*Nr3c1*	3.78 ± 0.26	2.99 ± 0.49	4.05 ± 0.17	3.36 ± 0.35
*Nr3c2*	8.54 ± 1.15	8.03 ± 0.87	8.32 ± 0.72	8.00 ± 0.48

Data are reported as mean ± SD, *n* = 8. AL: ad libitum; CO: corn oil; ER: energy restriction; *Nr3c1*, nuclear receptor subfamily 3 group C member 1; *Nr3c2*, nuclear receptor subfamily 3 group C member 2; SO: soybean oil; ΔCt: change in cycle threshold normalized to *β-actin*.

**Table A4:** ΔCt values for TH mediators.

	ΔCt			
Tissue/Gene	SO-AL	SO-ER	CO-AL	CO-ER
**Cerebellum**				
*Dio1*	11.95 ± 0.35	11.88 ± 0.42	12.02 ± 0.31	11.98 ± 0.45
*Dio2*	5.38 ± 0.26	5.19 ± 0.19	5.41 ± 0.26	5.29 ± 0.16
*Dio3*	13.04 ± 0.64	12.76 ± 0.28	13.32 ± 0.39	12.91 ± 0.34
*Mct8*	13.06 ± 0.80	13.07 ± 1.08	13.34 ± 1.09	14.09 ± 0.46
*Oatp1c1*	4.72 ± 0.28	4.78 ± 0.25	4.92 ± 0.37	5.27 ± 0.46
*Thra*	4.79 ± 0.09	4.73 ± 0.12	4.93 ± 0.09	4.78 ± 0.15
*Thrb*	9.65 ± 0.60	9.76 ± 0.18	10.10 ± 0.18	9.98 ± 0.24
**Cerebral Cortex**				
*Dio1*	14.00 ± 1.80	13.51 ± 1.21	13.19 ± 1.67	13.76 ± 0.87
*Dio2*	5.61 ± 0.18	5.35 ± 0.24	5.15 ± 0.14	5.26 ± 0.31
*Dio3*	11.03 ± 0.62	11.62 ± 0.67	10.95 ± 0.95	10.71 ± 1.13
*Mct8*	13.98 ± 0.39	13.99 ± 0.30	13.83 ± 0.46	13.69 ± 0.32
*Oatp1c1*	5.41 ± 0.12	5.50 ± 0.27	5.44 ± 0.27	5.84 ± 0.30
*Thra*	3.95 ± 0.09	3.84 ± 0.13	3.70 ± 0.15	3.61 ± 0.18
*Thrb*	6.91 ± 0.20	6.67 ± 0.26	6.62 ± 0.17	6.51 ± 0.17
**Hippocampus**				
*Dio1*	14.49 ± 1.24	13.93 ± 1.30	14.16 ± 1.23	14.28 ± 1.18
*Dio2*	5.53 ± 0.16	5.39 ± 0.18	5.50 ± 0.21	5.45 ± 0.26
*Dio3*	11.33 ± 0.62	11.02 ± 0.76	10.85 ± 0.97	10.97 ± 0.85
*Mct8*	12.49 ± 0.46	12.39 ± 0.52	12.32 ± 0.28	12.42 ± 0.42
*Oatp1c1*	5.47 ± 0.38	5.41 ± 0.31	5.42 ± 0.20	5.37 ± 0.22
*Thra*	2.82 ± 0.24	2.75 ± 0.22	2.67 ± 0.22	2.62 ± 0.19
*Thrb*	6.96 ± 0.17	6.75 ± 0.29	6.84 ± 0.55	6.63 ± 0.37
**Liver**				
*Dio1*	1.43 ± 0.37	0.68 ± 0.33	1.75 ± 0.36	0.87 ± 0.41
*Dio2**†*	11.85 ± 0.95	13.24 ± 3.13	13.27 ± 1.04	13.52 ± 1.55
*Dio3*	11.61 ± 0.98	12.13 ± 1.60	12.72 ± 1.04	12.37 ± 1.07
*Mct8*	8.33 ± 0.41	8.29 ± 0.36	8.77 ± 0.40	8.71 ± 0.36
*Oatp1c1**†*	16.03 ± 1.01	15.65 ± 0.91	16.43 ± 1.19	16.02 ± 0.43
*Thra*	6.13 ± 0.18	6.92 ± 0.14	6.25 ± 0.19	6.95 ± 0.24
*Thrb*	5.03 ± 0.18	4.32 ± 0.16	5.31 ± 0.14	4.61 ± 0.25

Data are reported as mean ± SD, *n* = 8. AL: ad libitum; CO: corn oil; *Dio1-3*: deiodinase 1-3; ER: energy restriction; *Mct8*: monocarboxylate transporter 8; *Oatp1c1*: solute carrier organic anion transporter family member 1C1; SO: soybean oil; *Thra*: thyroid hormone receptor alpha; *Thrb*: thyroid hormone receptor beta; ΔCt: change in cycle threshold normalized to *β-actin*. † signifies that the gene is not considered expressed in this tissue.

**Table A5:** qPCR Ct values for *β-actin*.

Tissue/Gene	SO-AL	SO-ER	CO-AL	CO-ER	*p*^a^	*p*^b^	*p*^c^
**Cerebellum**							
*β-actin*	14.92 ± 0.18	14.81 ± 0.12	14.81 ± 0.29	14.59 ± 0.20	0.73	0.15	0.75
**Cerebral Cortex**							
*β-actin*	14.11 ± 0.27	14.00 ± 0.12	14.21 ± 0.24	14.20 ± 0.21	0.72	>0.99	0.82
**Hippocampus**							
*β-actin*	14.26 ± 0.22	14.13 ± 0.19	14.06 ± 0.21	14.11 ± 0.19	0.57	0.95	0.20
**Liver**							
*β-actin*	16.83 ± 0.33	17.15 ± 0.25	16.55 ± 0.24	16.81 ± 0.25	0.11	0.22	0.17

Data are reported as mean ± SD, *n* = 8. Two-way ANOVA was performed on the Ct. Values in each tissue. *p*^a^ comparing SO with SO-ER; *p*^b^ comparing CO with CO-ER; *p*^c^ comparing SO with CO. AL: ad libitum; CO: corn oil; ER: energy restriction; SO: soybean oil; Ct: cycle threshold.
